# Atherogenic ApoB-dyslipidemia, atherosclerotic cardiovascular disease, cardiac dysfunction and remodeling in high-risk young women with and without polycystic ovary syndrome: A pilot study

**DOI:** 10.3389/fendo.2025.1520922

**Published:** 2025-09-09

**Authors:** Xiaoying Wu, Mich Wilke, Jesse Batara, Spencer Proctor, Melanie Cree, Mahua Ghosh, Paolo Raggi, Jonathon Windram, Harald Becher, Donna Vine

**Affiliations:** ^1^ PCOS Together Laboratory, University of Alberta, Edmonton, AB, Canada; ^2^ Women and Children’s Research Institute, Edmonton, AB, Canada; ^3^ Metabolic and Cardiovascular Disease Laboratory, University of Alberta, Edmonton, AB, Canada; ^4^ Division of Pediatric Endocrinology, Department of Pediatrics, University of Colorado Anschutz Medical Campus, Aurora, CO, United States; ^5^ Division Endocrinology and Metabolism, Faculty of Medicine and Dentistry, University of Alberta, Edmonton, AB, Canada; ^6^ Division Cardiology, Faculty of Medicine and Dentistry, University of Alberta, Edmonton, AB, Canada

**Keywords:** atherogenic dyslipidemia, atherosclerosis, plaque, cardiac function, cardiac remodeling, PCOS, ApoB-lipoproteins, remnant-cholesterol

## Abstract

**Introduction:**

Polycystic ovary syndrome (PCOS) is associated with increased cardiometabolic risk in young women of reproductive age. There are limited studies on atherogenic dyslipidemia, inclusive of triglycerides (TG), Apolipoprotein (apo) B-lipoproteins and remnant-cholesterol (C), atherosclerotic cardiovascular disease (ACVD), cardiac function and remodeling in young women with and without PCOS. The aim of this pilot study was to investigate the relationship of atherogenic dyslipidemia and other cardiometabolic risk factors with ACVD, cardiac function-remodeling in high-risk young overweight-obese PCOS women compared to non-PCOS and healthy-weight controls.

**Methods:**

Women with and without PCOS (non-PCOS control) aged 18 – 45 years who were overweight and obese (>25kg/m^2^) were matched for age and BMI, and by age with healthy-weight non-PCOS controls. PCOS diagnosis was based on Rotterdam criteria. Fasting lipids and non-fasting plasma lipid assessment included TG, remnant-C, total ApoB, ApoB48 and ApoB100. Carotid intimal-medial thickness (cIMT) and carotid plaque height were assessed, and cardiac function and remodeling were measured by 2-D ultrasound and 3D-echocardiography.

**Results:**

PCOS (n=48) and non-PCOS control overweight-obese age-BMI matched groups (n=19) were shown to have significantly higher fasting and non-fasting lipids including TG, remnant-C, total ApoB and ApoB48, compared to healthy-weight non-PCOS controls (n=10). PCOS and non-PCOS control overweight-obese groups had significantly higher SBP, DBP, cIMT and evidence of cardiac dysfunction and remodeling, with reduced Mitral E/A ratio, intraventricular (IV) relaxation time and increased Left ventricle (LV) end diastolic and systolic diameter, LV posterior wall thickness and IV septal thickness, compared to healthy-weight non-PCOS controls. Individuals with PCOS had significantly higher fasting plasma TG and remnant-C compared to the non-PCOS overweight-obese control group. The PCOS group tended to have 25% higher carotid plaque height, although this was not significant, compared to the non-PCOS overweight-obese control group. DBP, HOMA-IR and ApoB predicted 40% of the variability in cIMT and ApoB was shown to predict 14% of the variability in carotid plaque height, independent of age and BMI. A 1mg/ml increase in ApoB was associated with a 0.041mm increase in cIMT and a 0.75mm increase in carotid plaque height in all young women.

**Discussion:**

Our pilot results supports the potential of apoB-dyslipidemia, cIMT, carotid plaque height and left ventricular diastolic dysfunction and remodeling to be used in screening for CVD risk in high-risk populations such as overweight-obese women with and without PCOS. ApoB may be useful to predict atherosclerotic vascular burden and progression of cIMT and carotid plaque, and could be used to develop a female specific algorithm for ACVD risk in high-risk young women with and without PCOS.

## Introduction

Polycystic ovary syndrome (PCOS) is the most common reproductive-endocrine disorder affecting 5 - 15% of women globally and is associated with decreased quality of life and increased co-morbidities across the lifespan (1–11). In those with PCOS there is a high prevalence of risk factors for cardiovascular disease (CVD) including the metabolic syndrome, obesity, dyslipidemia, hypertension, insulin resistance, and diabetes (12–21). Increased prevalence of CVD has been reported in PCOS, inclusive of peripheral, cerebrovascular and coronary artery disease, venous thromboembolism and ischemic end stage CVD events (11, 14, 17, 22–28). Furthermore, women are known to experience gaps in cardiovascular care, particularly in early diagnosis, primary prevention and treatment (29, 30). We have reported CVD occurs in young women with a median age of 35 – 38 yrs, and occurs in PCOS 3 – 4 years earlier compared to those without PCOS (11). Our findings are consistent with other reports of increased CVD in young women with PCOS (<50 yrs of age) (11, 22). Screening of early atherosclerotic CVD (ACVD), atherogenic dyslipidemia and cardiac dysfunction and remodeling may help to identify those at high risk and in need for intervention to prevent progression of CVD (31–34). Subclinical ACVD inclusive of cIMT, endothelial dysfunction, hypertension, and cardiac dysfunction-remodeling have been reported to be more severe in young adolescents and women with PCOS (18 - 50yrs) (12, 13, 20, 27, 35–47). In young women with PCOS, cIMT is increased and positively associated with androgens, blood pressure and fasting dyslipidemia (12, 35, 39, 48, 49). Impaired left ventricular systolic and diastolic function, including lower left ventricular ejection fraction, impaired left ventricular global longitudinal strain and increased isovolumetric relaxation time, have been observed in PCOS compared to age-BMI matched controls (27, 28, 42). ACVD, left ventricular dysfunction and remodeling in asymptomatic young females is a predictor of future heart failure and CVD (34, 50, 51). Identifying early disease has prognostic value and can allow for early intervention and preventative measures to reduce the risk of future cardiovascular events (34, 50, 51).

We have shown young adolescents and women with obesity with and without PCOS have atherogenic dyslipidemia, including elevated triglycerides (TG) and Apolipoprotein (Apo) B-lipoproteins in the fasted and non-fasted state (13, 37, 52). These individuals have delayed clearance of TG and intestinal derived chylomicrons containing ApoB48 in the fed or non-fasting state (13, 37, 52). We have shown androgens are positively correlated with these higher levels of fasting and non-fasting plasma TG and chylomicron-ApoB48 (52). It is well established that TG-rich ApoB-lipoproteins and their cholesterol-dense remnants, including chylomicron remnants containing ApoB48 and very low-density lipoprotein (VLDL) remnants containing ApoB100, are atherogenic (53–55). These cholesterol dense ApoB-lipoprotein remnants, particularly ApoB48, accumulate in the arterial sub-endothelium and initiate atherogenesis (56–59). Elevated remnant-cholesterol (C) is a marker of these ApoB-lipoprotein remnants, and elevated remnant-C is established as a causal contributor to ACVD and ischemic vascular events (54, 60). Currently we have limited understanding of the relationship between ACVD, atherogenic dyslipidemia, and cardiac function-remodeling in young high-risk women with and without PCOS. The aim of this pilot study was to determine the association of apoB-lipoproteins and remnant-C with ACVD and cardiac function-remodeling in high-risk overweight-obese young women with PCOS compared to age-BMI matched non-PCOS and healthy-weight non-PCOS controls.

## Materials and methods

### Study design and population

We conducted an observational case-control study in high-risk overweight-obese women with PCOS (n=48) and without PCOS (non-PCOS control overweight-obese, n=19)) matched for age and body mass index (BMI). Healthy-weight non-PCOS controls (n=10) were also recruited as a reference group and matched for age. Participants were recruited from the greater community in Edmonton, Alberta from 2020 - 2022. Inclusion criteria: (1) patients between the ages of 18 – 45 years with and without PCOS, (2) BMI ≥ 25kg/m^2^, (3) elevated fasting plasma TG >1.7 mmol/L and/or apoB>0.8g/L. These individuals represent the most common high-risk cardiometabolic phenotype in those with and without PCOS (37, 61). Use of metformin was 27% and 10% in PCOS and non-PCOS groups, respectively. Anti-hypertensive medications were used in 15% of PCOS patients and not in the other groups. In all groups, 80 - 85% of participants were using an oral contraceptive and the use of oral contraceptive was not different between groups. The international guidelines to diagnose PCOS (Rotterdam criteria) were used based on two out of three of the following criteria: menstrual dysfunction, clinical and/or biochemical hyperandrogenism, and/or polycystic ovaries, and exclusion of other endocrine disorders (61). Exclusion criteria included pregnancy, lactation and use of fertility treatments. Ethics was obtained from the University of Alberta Human Research Ethics Board.

### Body composition

Body anthropometry (BMI, % body fat mass and % fat free mass) was determined using BodPod as previously described (52).

### Biochemical analyses

A venous fasting blood sample was collected following an overnight fast, and non-fasting blood was collected during the day (approximately 2 - 4hrs following a meal). Blood was centrifuged at 3000g for 10mins at 4 °C and then stored at -80 °C until further analysis. Fasting plasma and serum was used to measure Total Testosterone (T), free T, serum hormone binding globulin (SHBG), androstenedione, estradiol, dehydroepiandrostendione (DHEAS), glucose, insulin and lipids including total cholesterol (C), triglycerides (TG), LDL-C, HDL-C and total ApoB using standard accredited protocols (Alberta Precision Laboratories) (37). Non-HDL-C was calculated using TC-[HDL-C] and Remnant-C was calculated using TC-[LDL-C+HDL-C] (60). The free androgen index (FAI) was calculated: FAI = 100 (total T/SHBG) (62). The homeostasis model assessment index of insulin resistance (HOMA-IR) was calculated as fasting insulin (*μ*U/mL) × fasting glucose (mmol/L)/22.5 (63).

### ApoB-lipoproteins

ApoB48 and ApoB100 were quantified using a Western blot method as previously described (37, 52). In brief, total plasma proteins were separated on a 3% to 8% NUPAGE Tris-acetate polyacrylamide gel (Invitrogen, Carls- bad, CA) and then transferred onto a polyvinylidene difluoride membrane (0.45 mm, Immobilon PTM; Millipore, Billercia, MA). Membranes were incubated with a primary polyclonal antibody specific for ApoB (Santa Cruz Biotechnology, Dallas, TX) and a secondary antibody tagged with horseradish per- oxidase (Santa Cruz Biotechnology). ApoB48 and B100 bands were visualized by enhanced chemiluminescence (ECL Advance; Amersham Biosciences, Little Chalfont, UK) and quantified using linear densitometric comparison with a known mass of purified human ApoB48 and ApoB100 standards (37, 52).

### CVD Risk Scores

Framingham Risk Score (FRS) was calculated based on risk factors including age, Total-C, HDL-C level, systolic blood pressure, smoking status and diabetes (64). The 10-year ACVD risk (<5%: borderline risk, 5 - 7.5%%: intermediate risk, >7.5-20) was estimated based on the ACVD Pooled Cohort Equation validated for women which uses demographic age, sex, race and risk factors including BP, cholesterol, diabetes and smoking (29, 65). The American Society of Echocardiography ACVD risk stratification grade 0, I, II or III was calculated based on criteria of cIMT >1.5mm and/or carotid plaque height >1.5mm (31, 66).

### Atherosclerotic CVD, blood pressure and cardiac function

#### Carotid intima-media thickness and carotid plaque height

Carotid ultrasound imaging was performed in B-mode and analyzed using the Society of radiologist ultrasound recommendations and the ASE consensus (31). An experienced registered vascular technologist used 2-D high frequency vascular ultrasound to measure cIMT (L12 – 3 linear-array probe, frequency: 3e12 Mhz, axial resolution 0.8 mm; Epiq 7; Philips). cIMT was measured on the distal 1 cm of the far wall of the common carotid and is the distance between lumen-intima and media-adventitia interfaces. The digital recordings were stored in Digital Image and Communications in Medicine format for offline analysis using the semiautomated edge tracking Q-lab method (Philips, UK). These methods improve reproducibility and reduce reader variation of cIMT (31). Mean cIMT values for both right and left common carotid arteries were calculated using three separate measures for each side to reduce variability and error (31, 67). The presence or absence of carotid plaque, defined as more than 1.5 mm thickness of cIMT at the carotid bulb, and carotid plaque height were recorded as previously described (67–69).

#### Blood pressure, cardiac function-remodeling

Arterial blood pressure was measured by standard manual plethysmography at the brachial artery. 2-D and 3-D echocardiographic recordings were assessed to determine left ventricular mass index, interventricular septum thickness, left ventricular end-diastolic and left ventricular end-systolic diameter, left ventricular ejection fraction and parameters of diastolic left ventricular function including left atrial dimension, e’, and mitral E velocity and E/A ratio, as previously described (68, 70, 71). Relative wall thickness was determined as 2 x posterior wall thickness/left ventricular internal diameter at end- diastole, and was used with left ventricular Mass Index to determine left ventricular remodeling as normal, concentric or eccentric remodeling or hypertrophy (72). Left ventricular global longitudinal strain was measured using 2D-speckle-tracking echocardiography (70, 71). Echocardiographic images were obtained using Epiq scanner (Philips Medical systems), and left ventricular volume and ejection fraction analyses were performed off-line with a semi-automated tracing of end-systolic and end-diastolic endocardial borders from apical two-chamber and four-chamber images (Intellispace; Philips Medical Systems). The results were obtained using Simpson’s biplane method (70). Echocardiographic recordings were analysed using Qlab software (Philips) to measure left ventricular global longitudinal strain. Systolic left ventricular global longitudinal strain (%) was obtained by averaging the segmental strain curves at maximum instantaneous peak, as previously described (70).

### Statistical analysis

Descriptive statistics and normality tests were used for primary outcome variables and data expressed as mean ± SEM. One-way Analysis of Variance (ANOVA) followed by Bonferroni post-tests for multiple comparisons was used to test for a significant difference at p<0.05 between groups. Univariate linear regression was used to test the association of cIMT and carotid plaque height, with predictor variables of atherogenic dyslipidemia and cardiometabolic risk factors. Multiple linear regression analysis was employed to evaluate independent relationships between variables that were found to be statistically significant following correlation analysis, and backward stepwise elimination was used to determine predictor variables of cIMT and carotid plaque height. Multiple logistic regressions were used to test the association of presence of carotid plaque with cardiometabolic variables. A statistical significance of p<0.25 was used in selecting variables from univariate analyses to be included in the modelling, and a significant difference for a model used was p<0.05. Statistical analyses were performed using Graphpad Prism Version 9.4.1 (GraphPad Software, Inc., La Jolla, CA), SAS 9.4 and STATA 13.0 (Stata-Corp, College Station, TX).

## Results

### Anthropometry and biochemical parameters

There was no difference in total fat mass (%) or fat-free mass (%) between PCOS and non-PCOS control overweight-obese groups (**Table 1**). Both PCOS and non-PCOS control overweight-obese groups had significantly higher BMI, total fat mass and fat-free mass by 30 - 40% compared to the healthy-weight non-PCOS control group. The PCOS and non-PCOS control overweight-obese groups had elevated fasting insulin and HOMA-IR by approximately 60 - 70% compared to the healthy-weight non-PCOS controls, however this was only significantly different in those with PCOS compared to healthy-weight non-PCOS controls. Those with PCOS had a 2-fold higher free androgen index, and 60% higher free T and total T compared to both non-PCOS overweight-obese control and healthy-weight non-PCOS control groups, although due to high variability in data these differences were not statistically significant. SHBG was lower by 35% and 25% in those with PCOS and non-PCOS overweight-obese controls compared to the healthy-weight non-PCOS control group, however this was only statistically significantly in those with PCOS compared to healthy-weight non-PCOS controls (**Table 1**).

**Table 1:** Anthropometric and biochemical parameters of PCOS, Non-PCOS control and Healthy-Weight control groups.

	Healthy-Weight Control n=10	Non-PCOS Control n=19	PCOS n=48
Age (y)	30.80±2.51	34.05±1.48	33.81±0.81
BMI(kg/m^2^)	20.17±1.12	37.11±1.63*	38.34±1.92*
BSA (m^2^)	1.58±0.05	2.23±0.08*	2.19±0.04*
Total Fat Mass (%)	27.93±2.05	44.74±1.90*	44.66±1.17*
Total Fat-free Mass (%)	72.07±2.05	55.26±1.90*	55.34±1.17*
Glucose (mmol/l)	4.76±0.12	5.21±0.16	5.15±0.10
Insulin (pmol/l)	39.89±3.57	106.8±13.28	129.0±12.66*
HOMA-IR (mg/dL)	1.38±0.12	4.04±0.53	5.17±0.54*
Estradiol (pmol/l)	318.5±54.07	224.30±55.36	293.20±82.91
SHBG (nmol/L)	70.75±8.27	52.07±7.85	44.37±4.77*
Androstenedione (nmol/l)	8.69±0.96	7.67±0.77	9.35±0.60
Total T (nmol/l)	0.87±0.15	0.73±0.12	1.10±0.09
Free T (pmol/l)	9.08±1.92	10.11±2.03	16.13±1.51
DHEAS (umol/l)	6.33±0.89	4.43±0.76	6.81±0.06
FAI	1.66±0.48	1.87±0.42	5.10±2.14

Values are expressed as mean±SEM. PCOS, polycystic ovary syndrome; BMI, body mass index; BSA, body surface area, HOMA-IR, homeostasis model assessment insulin resistance; SHBG, sex hormone binding globulin; SEM, standard error of the mean. DHEAS, dehydroepiandrosterone sulfate, FAI, free androgen index. * P<0.05 indicates a statistically significance difference between groups.

### Fasting and non-fasting lipids and ApoB-lipoproteins

**Figure 1:**
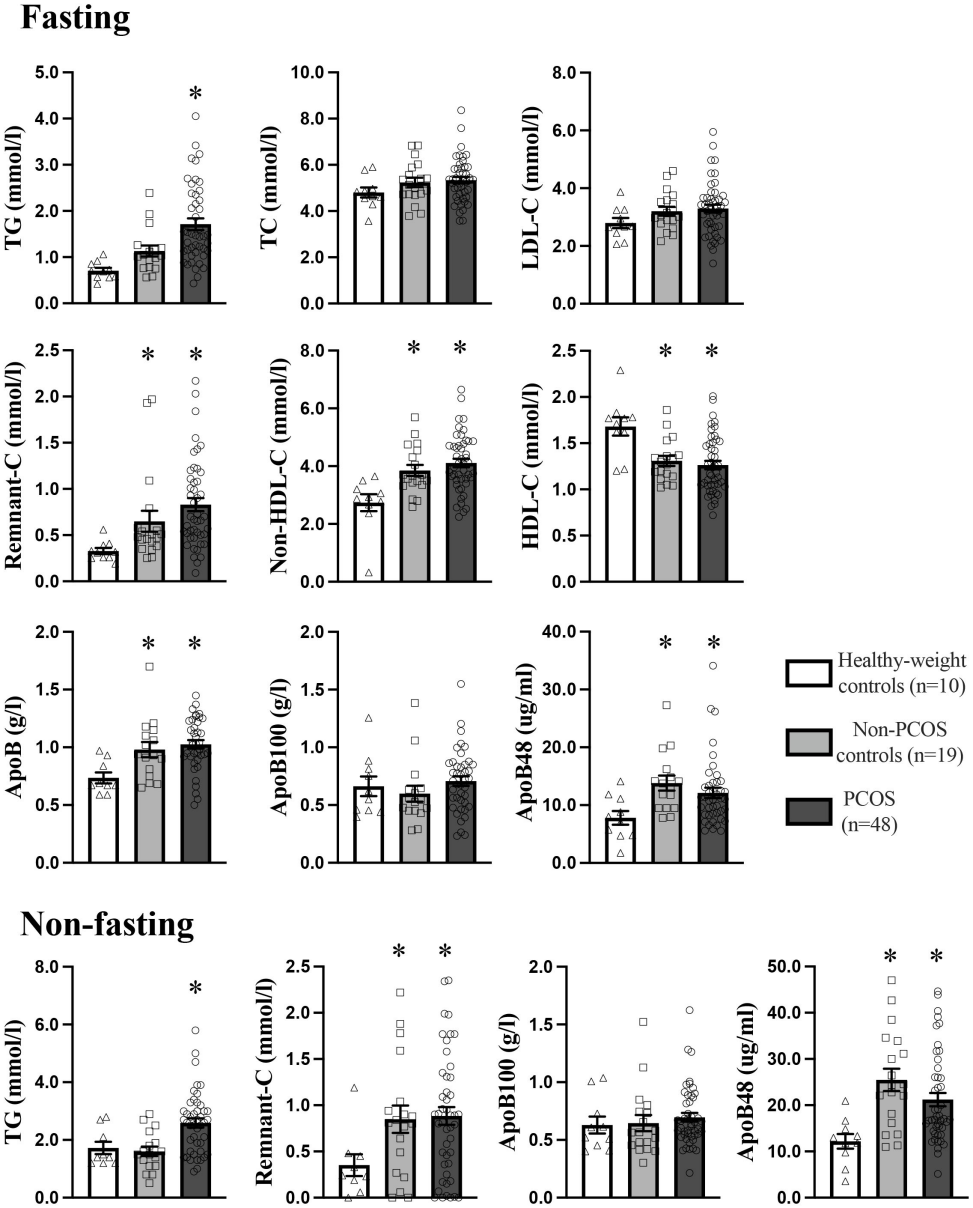
Fasting and non-fasting plasma lipid and apoB-lipoprotein profile in PCOS (n=48), non-PCOS control (19), and healthy-weight control (n=10) groups. Results are expressed as mean ± SEM. LDL-C, low-density lipoprotein cholesterol; HDL-C, high-density lipoprotein cholesterol; ApoB, apolipoprotein-B. * p<0.05 indicates a statistically significant difference and groups with a different symbol are significantly different.

Fasting non-HDL-C, remnant-C, and total apoB were significantly higher, and HDL-C was significantly lower in PCOS and non-PCOS overweight-obese groups compared to the healthy-weight non-PCOS control group (**Figure 1**). Those with PCOS had 50% and 30% higher fasting TG, which was significant compared to the healthy-weight non-PCOS and non-PCOS overweight-obese controls, respectively. Those with PCOS had 50% and 20% higher remnant-C compared to the healthy-weight non-PCOS and non-PCOS overweight-obese controls, and this was significantly different compared to the healthy-weight non-PCOS controls. Overall, these results highlight a clinically significant exacerbation of these fasting lipids in individuals with PCOS. Fasting and non-fasting ApoB48 concentration, representing intestinal chylomicrons, were 30 - 50% higher in PCOS and non-PCOS overweight-obese groups, and this was significant compared to the healthy-weight non-PCOS control group (**Figure 1**). There were no differences in fasting or non-fasting ApoB100 concentrations, representing hepatic derived lipoproteins, between groups consistent with our previous findings (37, 52). Fasting and non-fasting remnant-C was significantly higher by 2-fold in PCOS and non-PCOS overweight-obese individuals, compared to the healthy-weight non-PCOS control group.

### Blood pressure and cardiac function-remodeling

SBP and DBP were significantly higher by 20 - 30% in PCOS and non-PCOS overweight-obese control groups compared to healthy weight non-PCOS controls, consistent with other studies in overweight-obese females with and without PCOS (**Table 2**) (20, 22). PCOS and non-PCOS overweight-obese groups had specific impairments in cardiac function that were significantly different compared to healthy-weight non-PCOS controls. Mitral E/A ratio was significantly lower by 30% and 20% in PCOS and non-PCOS overweight-obese groups, respectively, compared to healthy-weight non-PCOS controls. Both PCOS and non-PCOS overweight-obese groups had a significantly prolonged intraventricular relaxation time by 20% compared to the healthy-weight non-PCOS controls. Those with PCOS had significantly higher A peak velocity by 20% compared to non-PCOS overweight-obese controls and healthy-weight non-PCOS controls. PCOS and non-PCOS overweight-obese groups had significantly increased left ventricular remodeling compared to healthy-weight non-PCOS controls. Left ventricular posterior wall and interventricular septum thickness and left ventricular end diastolic and systolic diameters were increased by 15 - 25% in PCOS and non-PCOS overweight-obese groups compared to healthy-weight non-PCOS controls. Left ventricular mass index was increased by 20% in PCOS and non-PCOS overweight-obese groups, and this was significantly higher in the PCOS group compared to the healthy-weight non-PCOS control group. Left ventricular concentric remodeling was observed at a 30% higher rate in those with PCOS compared to the non-PCOS overweight-obese and healthy-weight non-PCOS control groups. Left ventricular global longitudinal strain was impaired by 5% in PCOS and non-PCOS overweight-obese groups compared to healthy-weight non-PCOS controls, however this did not reach statistical significance. Left ventricular posterior wall thickness and A peak ratio were shown to be positively associated with cIMT, and Mitral E/A was negatively associated with cIMT in all groups (**Table 3**).

**Table 2:** Blood Pressure and cardiac function in PCOS, Non-PCOS control and Healthy-Weight control groups.

	Healthy-Weight control n=10	Non-PCOS control n=19	PCOS n=48
SBP (mmHg)	108.3±2.58	129.4±3.13*	128.3±1.65*
DBP (mmHg)	66.80±2.28	81.84±3.22*	78.43±1.59*
Heart Rate (bpm)	74.30±3.43	75.11±2.36	77.81±2.19
LV Mass Index (g/cm^2^)	50.33±2.83	62.14±2.31	64.78±1.99*
LV End Diastolic (cm)	4.29±0.15	4.83±0.13*	4.77±0.06*
LV End Systolic (cm)	2.65±0.11	3.12±0.11*	3.08±0.07*
LVPW Thickness (cm)	0.69±0.04	0.86±0.02*	0.84±0.02*
IVS Thickness (cm)	0.70±0.03	0.84±0.03*	0.84±0.02*
Left Atrial Dimension (cm)	0.25±0.11	0.31±0.14*	0.28±0.11
RWT (cm)	0.33±0.02	0.36±0.01	0.35±0.008
Fractional Shortening (%)	38.48±2.79	35.44±1.52	35.55±1.09
LVEF (%)	64.20±0.47	62.21±0.75	61.69±0.49
LVGLS (%)	22.61±0.49	21.52±0.31	21.73±0.18
E Peak velocity (Rate) (m/s)	0.85±0.03	0.83±0.04	0.82±0.02
A Peak velocity (Rate) (m/s)	0.47±0.02	0.58±0.03	0.65±0.02*
e’m Velocity (Rate) (m/s)	0.13±0.008	0.13±0.009	0.12±0.006
Average E/e’	6.59±0.42	6.91±0.32	7.05±0.28
Mitral E/A Ratio	1.85±0.14	1.44±0.09*	1.26±0.05*
IV Relaxation Time (ms)	64.44±2.94	79.47±3.10*	77.45±2.18*
IV Contraction Time (ms)	64.44±1.76	68.42±3.69	67.23±1.87
LV Concentric Remodeling (%)	10	10.5	14.5

Values are expressed as mean±SEM. SBP, systolic blood pressure; DBP, diastolic blood pressure; LV, left ventricle; PW, posterior wall; IVS, interventricular septum thickness; RWT, relative wall thickness; EF, ejection fraction; GLS, global longitudinal strain. E, early diastolic inflow velocity; A, late diastolic inflow velocity; e’m, myocardial early diastolic annular velocity; IV, isovolumetric. * P<0.05 indicates a statistically significance difference between groups.

**Table 3:** Univariate linear regression of predictive variables with cIMT in PCOS, non-PCOS and Healthy-Weight Controls.

	r	Adjr^2^	P
Age	0.53	0.27	<0.001
BMI	0.23	0.05	0.045
SBP	0.33	0.11	0.004
DBP	0.38	0.14	<0.001
HOMA-IR	0.15	0.02	0.220
T	-0.05	-0.002	0.650
FreeT	-0.01	-0.001	0.930
FAI	0.12	0.01	0.340
Fasting			
TG	0.32	0.96	0.004
TC	0.12	0.01	0.300
LDL-C	0.06	0.004	0.570
HDL-C	-0.23	-0.053	0.043
Non-HDL-C	0.16	0.026	0.090
Remnant-C	0.15	0.022	0.190
ApoB	0.35	0.12	0.005
Apo48	0.017	0.0003	0.890
ApoB100	0.004	0.00002	0.970
Non-Fasting			
TG	0.21	0.044	0.070
ApoB48	0.08	0.006	0.510
ApoB100	-0.007	-0.000049	0.950
Remnant-C	0.14	0.019	0.229
Plaque Height	0.16	0.026	0.169
Cardiac Outcomes			
LVPW	0.39	0.15	0.0004
LA	0.05	0.0025	0.610
A	0.32	0.10	0.004
Mitral E/A	-0.39	-0.15	0.0005
FRS	0.48	0.23	0.0001

### Subclinical ACVD assessment: cIMT and carotid plaque

**Figure 2:**
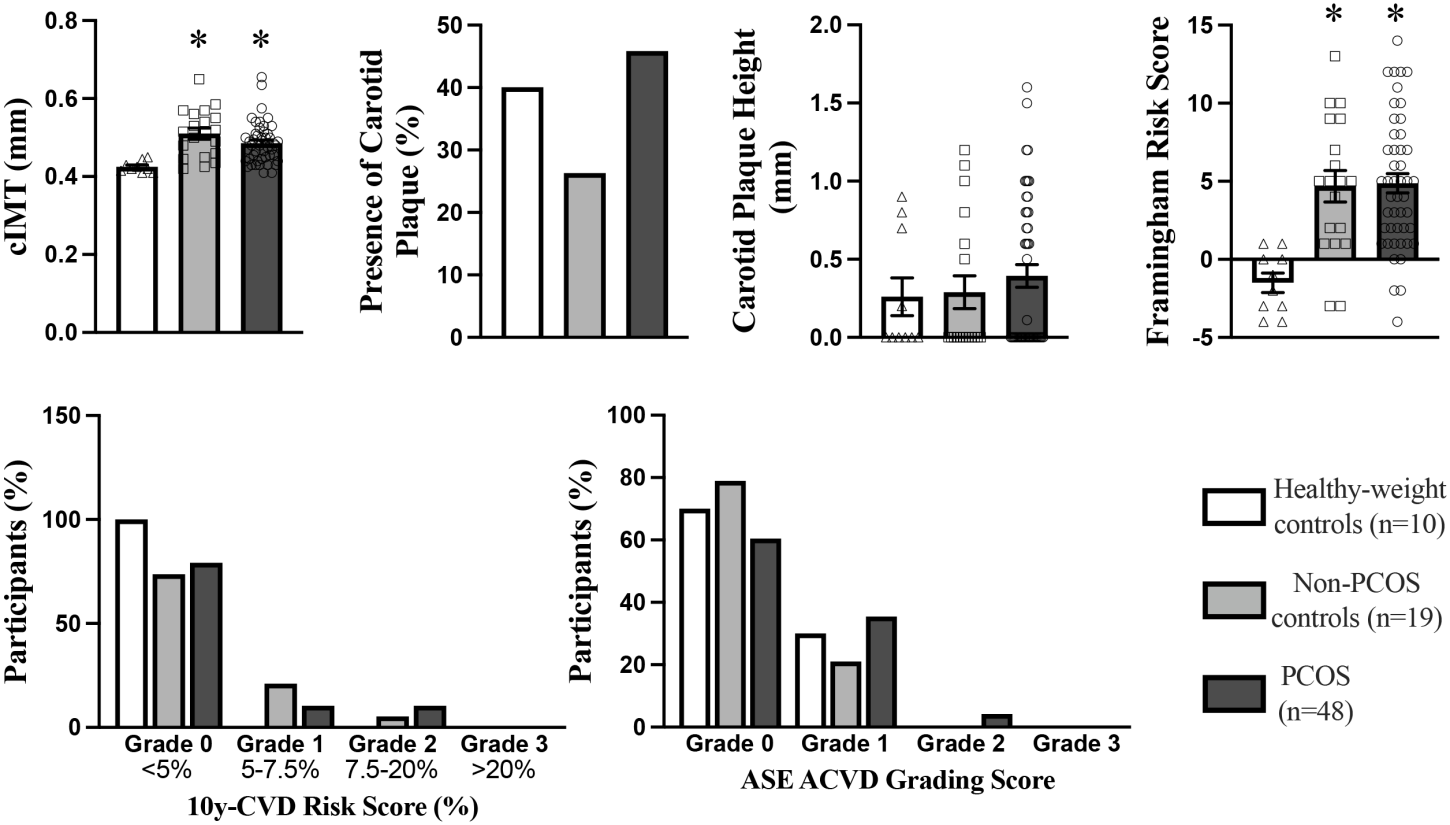
Carotid Intima-Media Thickness, Carotid Plaque, Framingham Risk Score 10yr CVD Risk Score, and ASE Grading CVD Risk Score in PCOS (n=48), non-PCOS control (n=19), and Healthy-weight controls (n=10). cIMT, carotid intima-media thickness. ASE, American Society of Echocardiography. *p<0.05, indicates a statistically significant difference between groups.

cIMT was significantly higher by 10 - 15% in the PCOS and non-PCOS overweight-obese groups compared to the healthy-weight non-PCOS control group (**Figure 2**). In univariate analyses age, BMI, SBP, DBP, TG, ApoB were positively correlated, whilst HDL-C was negatively correlated with cIMT (**Table 3**). Stepwise multivariable linear regression analyses showed that age, DBP and ApoB predicted 37.8% of cIMT (**Table 4**). An increase in age by 1yr was associated with an increase in cIMT of 0.04mm, whereas an increase in DBP by 1 mmHg was associated with an increase in cIMT of 0.014 mm (**Table 4**). A 1mg/ml increase in ApoB was associated with an increase in cIMT of 0.041 mm (**Table 4**). After adjusting for age and BMI, DBP and HOMA-IR significantly predicted 40% of cIMT.

**Table 4:** Multivariable linear Regression of predictor variables with cIMT in PCOS, non-PCOS and Healthy-Weight Controls.

Variable	*Adj R^2^*	B (95% CI)	P
*cIMT*			
Model 1	0.377		
Age		0.004 (0.100-0.289)	0.0001
DBP		0.0014 (0.001-0.003)	0.015
ApoB		0.051 (-0.002-0.114)	0.052
Model 2: adjusted for Age and BMI	0.402		
Age		0.004 (0.002-0.006)	<0.0001
BMI		-0.001 (-0.002-0.001)	0.475
DBP		0.002 (0.0004-0.003)	0.007
HOMA-IR		0.004 (0.0003-0.007)	0.035
ApoB		0.041 (-0.014-0.095)	0.144

The prevalence of carotid plaque was 45%, 26% and 40% in women with PCOS, non-PCOS overweight-obese controls and healthy-weight non-PCOS controls, respectively (**Figure 2**). Although not statistically significant, carotid plaque height was 30% higher in those with PCOS compared to non-PCOS overweight-obese controls and healthy-weight non-PCOS controls, suggesting a tendency for exacerbated development of carotid plaque lesions in those with PCOS (**Figure 2**). Univariate analyses showed carotid plaque height was positively correlated with predictor variables including age, fasting total-C, LDL-C and ApoB, and non-fasting remnant-C (**Table 5**). In multivariate linear regression modelling, age, fasting apoB and non-fasting remnant-C were shown to predict carotid plaque height and accounted for 20% of the variability in carotid plaque height (**Table 6**). When adjusting for age and BMI, ApoB predicted 14% of the variability in carotid plaque height and a 1mg/ml increase in total ApoB was associated with a 0.75mm increase in carotid plaque height.

**Table 5:** Univariate linear regression of predictor variables with carotid plaque height in PCOS, non-PCOS and Healthy-Weight Controls.

	r	Adjr^2^	P
Age	0.23	0.05	0.050
BMI	0.10	0.01	0.381
SBP	0.12	0.01	0.291
DBP	0.03	0.001	0.772
HOMA-IR	0.04	0.002	0.767
T	-0.07	-0.005	0.543
Free T	0.04	0.002	0.719
FAI	-0.08	-0.006	0.511
Fasting			
TG	0.14	0.02	0.220
TC	0.26	0.07	0.025
LDL-C	0.26	0.07	0.021
HDL-C	-0.039	-0.001	0.739
Non-HDL-C	0.19	0.04	0.090
Remnant-C	0.10	0.01	0.387
ApoB	0.37	0.14	0.003
Apo48	-0.02	-0.0004	0.872
ApoB100	0.097	0.009	0.431
Non-Fasting			
TG	0.06	0.004	0.631
ApoB48	-0.05	-0.002	0.687
ApoB100	-0.06	-0.004	0.624
Remnant-C	0.28	0.079	0.014
Cardiac Outcomes			
LVPW	0.16	0.03	0.165
LA	0.18	0.03	0.123
A	0.17	0.03	0.139
Mitral E/A	-0.24	0.06	0.041
FRS	0.17	0.03	0.137

**Table 6:** Multivariable linear Regression of predictor variables with carotid plaque height in PCOS, non-PCOS and Healthy-Weight Controls.

Variable	*Adj R^2^*	B (95% CI)	*P*
Carotid Plaque Height			
Model 1	0.200		0.001
Age		0.02 (-0.001-0.032)	0.067
ApoB		0.46 (-0.03-0.950)	0.065
Non-Fasting RC		0.20 (-0.002-0.03)	0.074
Model 2: adjusted for age and BMI	0.140		0.007
Age		0.02(-0.002-0.03)	0.074
BMI		-0.0.1 (-0.02-0.01)	0.354
ApoB		0.75 (0.26-1.23)	0.003

### CVD Risk Scores

Framingham Risk Score was significantly higher by 4-fold in PCOS and non-PCOS overweight-obese groups compared to healthy-weight non-PCOS controls (**Figure 2**). The Framingham Risk Score was positively correlated with cIMT (**Table 3**). The intermediate and tertiary 10-year ACVD risk scores of 5 - 7.5% and >7.5-20% tended to be more prevalent in those with PCOS and non-PCOS overweight-obese groups by 10 - 20% (**Figure 2**), compared to healthy-weight non-PCOS controls. The PCOS group tended to have a 2-fold higher prevalence rate of intermediate risk score (>7.5-50%) compared to the non-PCOS overweight-obese control group (**Figure 2**). The American Society of Echocardiography ACVD Risk scores were tended to be ranked higher in those with PCOS compared to non-PCOS overweight-obese control and healthy-weight non-PCOS control groups (**Figure 2**). The American Society of Echocardiography ACVD Risk score uses the criteria of cIMT >1.5mm or plaque height >1.5mm to grade lesions and ACVD risk (66). Using these criteria 10 - 20% of those with PCOS were re-classified to a higher ACVD risk score of Grade I compared to non-PCOS overweight-obese controls and healthy-weight non-PCOS controls (66). Only those with PCOS has lesions re-classified to Grade II for ACVD risk, with 6% of individuals with PCOS classified with Grade II lesions (**Figure 2**).

## Discussion

Our pilot findings are the first to show young high-risk overweight-obese women with and without PCOS have impaired plasma TG and ApoB-lipoprotein metabolism and this is associated with increased cIMT. Those with PCOS and non-PCOS overweight-obese controls were shown to have elevated blood pressure, impaired cardiac left ventricular diastolic function, left ventricular hypertrophy, and higher CVD risk scores. Our data are consistent with our previous studies, suggesting altered ApoB-TG-rich lipoprotein metabolism may be an important indicator of early atherosclerotic vascular burden in young high-risk overweight-obese women with and without PCOS (37, 52).

ApoB48 is a marker of intestinal chylomicrons and upon lipolysis these form cholesterol dense chylomicron remnants. These chylomicron remnants are part of the pool of circulating remnant-C (60). Remnant-C is an established causal CVD risk factor and is predictive of end-stage ischemic CVD events, including cerebral and myocardial infarction (37, 52, 60, 73). Total-C, LDL-C and ApoB100 were not different between groups and appear to have limited clinical value to detect early dyslipidemia in young women (13, 37, 52). Rather, our data shows impairment in chylomicron-apoB48, TG and remnant-C are potentially better markers of early atherogenic ApoB-dyslipidemia in young high-risk overweight-obese women with and without PCOS. Although not statistically significant, those with PCOS had 25% higher fasting and non-fasting plasma TG, fasting remnant-C, carotid plaque height and higher ACVD scores, compared to the non-PCOS overweight-obese controls. These findings may represent a clinically significant finding for prediction of CVD risk, however further larger studies are required to corroborate these pilot findings.

Systolic and diastolic blood pressure were significantly higher in age-BMI matched PCOS and non-PCOS overweight-obese groups compared to healthy-weight non-PCOS controls. Other studies have reported blood pressure is higher in age-BMI matched individuals with PCOS compared to non-PCOS controls, however we found no difference which may be due to our small sample size (18, 20). We observed mild hypertension was observed in 50% of those with PCOS and non-PCOS overweight-obese groups (74). Mild hypertension is common in high-risk young women with overweight-obesity and PCOS but may often be undetected and under-treated in the primary care setting (18, 20, 29, 75, 76). Furthermore, reports have highlighted that PCOS patients are often not referred to diet-lifestyle therapy or consulted on options for anti-hypertensive treatments (29, 75, 76).

Both cIMT and carotid plaque height measure early subclinical ACVD and both are recommended to be used in young high-risk individuals rather than measurement of coronary calcium score (46, 77–82). This is because coronary calcium score is a marker of advanced lesions in older populations aged >55 yrs (46, 78–81). cIMT can be a marker of non-atherosclerotic arterial hypertrophy and is a subclinical predictor of risk of myocardial infarction, stroke and coronary heart disease (31, 83, 84). Consistent with other studies, we have shown overweight-obese young women aged 18 - 45yrs with and without PCOS have 15% higher cIMT, compared to healthy-weight non-PCOS controls (12, 35, 39, 85). Interestingly, healthy-weight non-PCOS controls also had the presence of cIMT and carotid plaque, which has been observed in young women, and is considered normal vascular aging associated with early subclinical stages of atherosclerosis (35, 48, 49, 82). Age and BMI are associated with increasing carotid intimal thickening and our data consistently shows age and BMI are significant predictors of cIMT in young women (31, 48, 86, 87). Increased vascular thickening is proposed as an adaptation to hypertension, endothelial dysfunction and inflammation observed in PCOS and obesity (38, 40, 45, 87, 88). We have shown age, DBP and fasting ApoB predict 37% of the variance in cIMT, consistent with a previous report in young women showing ApoB was positively correlated with cIMT (48). Our pilot data found that androgens were not associated with cIMT and this is in contrast to other reports of a positive association of androgens with vascular thickening in those with PCOS and obesity (45, 48, 49, 88–96). Our findings may reflect the use of anti-hypertensives and insulin-sensitizers in our patients with PCOS and these have been shown to be protective in vascular remodeling (97, 98).

We have shown a 1mg/ml increase in ApoB predicted a 0.75mm increase in carotid plaque height. Total ApoB reflects both hepatic VLDL-ApoB100 and intestinal chylomicron-ApoB48 TG rich lipoproteins and their cholesterol-dense remnants in the circulation. Increased ApoB-TG rich lipoproteins lead to increased remnant-C and this is a causative risk factor in atherosclerotic vascular disease (60). Therefore our data shows ApoB and remnant-C may be useful markers of plaque development in young women (29). The significance of screening for carotid plaque is that it is a direct measure of atherosclerosis and coronary vascular atherosclerotic burden (84, 99). Moreover carotid plaque has been shown to be a predictor of long-term CVD events including stroke resulting from luminal stenosis and plaque rupture (84, 99). Carotid plaque height has also been shown to more accurately predict ischemic coronary artery disease compared to cIMT in asymptomatic individuals (86, 87, 100–103). Asymptomatic individuals would include young individuals who do not present with symptoms of vascular disease or cardiac dysfunction (86, 87, 100–103). We have shown individuals with PCOS tend to have higher ACVD risk scores using the American Society of Echocardiography guidelines which is based on both cIMT and carotid plaque height measurements, however this is a limited sample size and data would need to be validated in a larger sample size or population (31). We found the Framingham Risk Score was positively associated with cIMT in young women. The Framingham Risk Score was significantly higher in those with PCOS and overweight-obese non-PCOS controls. The 10 year-ACVD risk scores tended to be higher in PCOS and non-PCOS overweight-obese groups, compared to the healthy-weight non-PCOS controls. Those with PCOS had a 50% increase in 10yr-ACVD risk score to intermediate risk score >7.5-20%, compared to the non-PCOS overweight-obese control group and these findings would need to be validated in a larger sample size. Although the actual scores for the Framingham Risk Score and 10-yr ACVD risk are considered low, the reference population used for these scores is older females aged 50 – 75 yrs (29, 64, 65, 104). Therefore, our results suggest that even a low risk ACVD score may be used to indicate increased atherogenic risk (29, 64, 65, 104) and the generation of a Framingham Risk Score or 10 year-ACVD risk score may be a useful tools to predict risk in young women (11, 40, 105–113). It is recommended in asymptomatic individuals with an increased 10yr-ACVD risk score that progression of vascular disease be monitored and that preventive therapies be implemented (29, 31, 33, 84). For a low (<5%) 10yr-ACVD risk score it is recommended diet and lifestyle factors be discussed with patients (29). For borderline risk (5 - 7.5%) and intermediate risk (7.5 - 20%), it is recommended ACVD risk enhancers be factored into shared decision-making on medications to address dyslipidemia and blood pressure following existing guidelines (29). A cIMT and carotid plaque height >1.5 mm are considered clinically significant in development of diffuse and protuberant atherosclerosis in those <65 years of age, and would prompt discussions with patients on cardiometabolic risk factor reduction following current guidelines (31, 114). Our pilot results highlight that in asymptomatic young overweight-obese females these assessments may help to identify individuals at high ACVD risk (29, 31, 33, 84).

Early asymptomatic impairment of left ventricular function and remodeling is associated with hypertension and atherosclerotic heart disease (34, 50, 51). These measurements are used to identify high-risk individuals and to initiate preventive care to avert overt heart failure and CVD events (34, 50, 51). Our results are consistent with studies that show left ventricular remodeling and cardiac dysfunction in age-BMI matched overweight-obese young women with and without PCOS (27, 28, 42, 47, 115–117). We have shown left ventricular posterior wall thickness is positively correlated with cIMT, reflecting the relationship of carotid hypertrophy with left ventricular remodeling (31, 87, 118, 119). We have shown mitral E/A ratio was inversely correlated with cIMT. Mitral E/A ratio represents impaired relaxation and diastolic dysfunction, and both cIMT and mitral E/A ratio are associated with hypertension and left ventricle hypertrophy (47). Left ventricular global longitudinal strain is a very early marker of left ventricular systolic dysfunction, and can be detected when left ventricular ejection fraction is normal (27, 42, 71). Although our pilot data was not significant, left ventricular global longitudinal strain was decreased by approximately 5% in young women with overweight-obesity with or without PCOS. Other studies have shown a significant reduction of 5 - 10% in those with PCOS compared to age-BMI matched non-PCOS controls (27). The use of left ventricular global longitudinal strain may be useful in early screening and monitoring of progressive left ventricular strain in young women who are at high-risk such as those with PCOS and overweight-obesity (42, 120).

The limitations of our data is that it is a pilot study with a small sample size, and data needs validating in a larger sample size and population, and it is a single center study. We did not include healthy-weight PCOS patients due to small numbers and we did not account for PCOS phenotype which may impact cardiac function and CVD risk (47, 49, 89, 121, 122). We did not find a significant relationship between androgens (free T or FAI), ACVD and cardiac dysfunction which is in contrast to hyperandrogenemia being implicated in the pathophysiology of increased CVD risk in those with PCOS (119, 123). Rather our data was consistent with overweight-obesity contributing significantly to CVD risk in PCOS (2, 3, 7, 14). We did not assess microvascular disease, however impaired endothelial function has been reported previously in young women with PCOS and obesity (38, 40, 44, 45, 124, 125). Other tests for CVD or cardiac function were not examined such as Stress Testing or Computed Tomography. Although our participants were young women and likely to be at lower risk for Acute Coronary Syndrome or Obstructive Coronary Disease and Spontaneous Coronary Artery Dissection, these tests could be done in future studies (29). The strengths of our findings include ‘a priori’ testing of the relationship between fasting and non-fasting remnant-C and apoB-lipoproteins that are early markers of impaired lipid metabolism and are causal factors in atherogenesis (13, 54, 60). In addition, our findings are consistent with recommendations for combined assessment of cIMT and carotid plaque height for subclinical atherosclerotic CVD screening in young individuals (31, 84, 86). Further studies are needed in larger cohorts to test the feasibility of ACVD screening and to understand the natural history of apoB-dyslipidemia, ACVD and cardiac dysfunction in young women. Furthermore, female-specific CVD ‘risk-enhancement’ factors such as PCOS, menstrual dysfunction and pregnancy complications such as pre-eclampsia, have been recommended to be taken into consideration when estimating CVD risk in young women (29, 32, 126). Therefore, larger studies with more comprehensive data sets are required to develop an algorithm to contribute to evidence-based screening of ACVD risk in young women with and without PCOS (31, 33, 102).

The findings of this pilot study have shown early impaired TG and apoB-lipoprotein metabolism in overweight-obese women with and without PCOS, and this was associated with increased cIMT, and left ventricular diastolic dysfunction and hypertrophy. CVD ischemic events have been reported to be increased in young women with PCOS therefore early ACVD and cardiac function screening may be warranted, however larger studies are needed to determine the cost-benefit of these assessments (11). Future studies could include these assessments and be used to develop an algorithm to determine plaque burden, predict ischemic CVD events and to identify high-risk young women who require preventative intervention therapies (29–33, 127). In conclusion, these findings support the need for larger trials on the potential utility of screening for ACVD, cardiac dysfunction and impaired apoB-metabolism in the primary care management of CVD risk in young high-risk overweight-obese women with and without PCOS.

## Data Availability

The raw data supporting the conclusions of this article will be made available by the authors, without undue reservation.
